# Association of HLA-G Expression, Its Genetic Variants and Related Neuro-Immunomodulation with Characteristics of Bladder Carcinoma

**DOI:** 10.3390/cancers16223877

**Published:** 2024-11-20

**Authors:** Vladimira Durmanova, Iveta Mikolaskova, Eszter Zsemlye, Agata Ocenasova, Helena Bandzuchova, Magda Suchankova, Boris Kollarik, Patrik Palacka, Milan Zvarik, Maria Bucova, Luba Hunakova

**Affiliations:** 1 Institute of Immunology, Faculty of Medicine, Comenius University in Bratislava, 811 08 Bratislava, Slovakia; vladimira.durmanova@fmed.uniba.sk (V.D.); mikolaskova6@uniba.sk (I.M.); agata.ocenasova@fmed.uniba.sk (A.O.); magda.suchankova@fmed.uniba.sk (M.S.); maria.bucova@fmed.uniba.sk (M.B.); 2 National Transplant Organisation, 831 01 Bratislava, Slovakia; helena.bandzuchova@nto.sk; 3 Department of Urology, Saints Cyril and Methodius Hospital, University Hospital Bratislava, 851 07 Bratislava, Slovakia; 4 2nd Department of Oncology, Faculty of Medicine and National Cancer Institute, Comenius University, Klenova 1, 833 10 Bratislava, Slovakia; patrik.palacka@nou.sk; 5 Cancer Research Institute, Biomedical Research Center of the Slovak Academy of Sciences, 814 39 Bratislava, Slovakia; 6 Department of Nuclear Physics and Biophysics, Faculty of Mathematics, Physics and Computer Science, Comenius University in Bratislava, 842 48 Bratislava, Slovakia; milan.zvarik@fmph.uniba.sk

**Keywords:** HLA-G, bladder cancer, mRNA expression, heart rate variability (HRV), inflammation

## Abstract

Background: Human leukocyte antigen G (HLA-G) is an immune checkpoint molecule with immunosuppressive and anti-inflammatory activities. It belongs to class I non-classical major histocompatibility complex molecules and has been upregulated in various cancer types. In bladder cancer (BC) tumors, the association of HLA-G with cancer progression has to be explained. Methods: A total of 89 BC patients and 74 control subjects were genotyped for the HLA-G 14 bp ins/del polymorphism. In urine cell samples, HLA-G mRNA expression was analyzed using real-time PCR. Soluble HLA-G (sHLA-G) serum levels were measured by ELISA. The associations between the HLA-G 14 bp ins/del polymorphism, HLA-G mRNA expression, and/or sHLA-G levels and selected variables including tumor grade, disease stage, body mass index, and heart rate variability (HRV) parameters were evaluated. Results: The protective HLA-G 14 bp ins/ins genotype under the recessive genetic model was associated with lower HLA-G mRNA expression in the BC group (*p* = 0.049). Significantly higher HLA-G mRNA expression was detected in patients with pT2 + pT3 as compared to those with pTa + pT1 stages (*p* = 0.0436). Furthermore, higher HLA-G mRNA expression was observed in high-grade muscle-infiltrating BC (MIBC) than in the low-grade non-MIBC group (*p* = 0.0365). Patients with a level of sHLA-G above 29 U/mL had shorter disease-free survival than patients with lower sHLA-G levels. Furthermore, the opposite HRV correlations with sHLA-G levels in BC patients as compared to controls probably reflect the different roles of HLA-G in health and cancer. Conclusions: Our results suggest the impact of the HLA-G 14 bp ins/del variant, HLA-G expression, and autonomic nervous system imbalance on advanced stages of BC.

## Simple Summary

Human leukocyte antigen G (HLA-G) is an immunosuppressive molecule that is often found at higher levels in various cancers, including bladder cancer (BC). We looked at 89 BC patients and 74 control subjects to understand how HLA-G is linked to BC progression. We examined specific genetic variations and measured HLA-G levels in urine and blood samples, also considering factors such as tumor stage, body mass index, and heart rate variability (HRV). We found that a particular genetic variation was associated with lower HLA-G mRNA levels in the urine of BC patients. Conversely, higher HLA-G mRNA levels were found in patients with advanced stages of cancer. Moreover, patients with higher blood levels of HLA-G had shorter disease-free survival. HRV was linked to HLA-G levels differently in healthy people compared to those with BC. These results suggest that HLA-G plays a role in BC progression and may be influenced by genetic factors and the body’s stress response.

## 1. Introduction

Bladder cancer (BC), mostly represented by urothelial carcinoma (UC), belongs to the most common neoplasms. Its worldwide incidence was estimated to be around 3% in 2020. It has a high rate of recurrence (48–71% of patients) and a 5% rate of death [1]. Tumors that do not invade the muscle layer of the bladder are classified as Ta and T1. They are treated through transurethral resection of bladder tumor (TURBT), with subsequent chemotherapy or intravesical immunotherapy with Bacillus Calmette–Guerin (BCG). Tumors that invade the muscle of the bladder or beyond are classified as T2, T3, and T4 and their treatment involves, in most cases, radical cystectomy [2]. Ta and T1 stage tumors are termed non-muscle-invasive bladder cancer (NMIBC); however, other tumor stages are classified as muscle-invasive bladder cancer (MIBC) [3]. 

The main exogenous risk factors for BC are smoking and exposure to environmental carcinogens. Smoking is the primary cause of BC with a lifetime absolute risk of 7.1% among white men aged 50 who are current smokers [4]. The endogenous risk factors for BC development include genetic susceptibility. Nowadays, more than 197 genes have been associated with the risk of BC. They include genes associated with cell metabolism, DNA repair, cell cycle pathways, and immune regulation [5,6]. 

The non-classical human leukocyte antigen G (HLA-G) is an immune checkpoint molecule with suppressive activities. Its predominant expression occurs in the extravillous cytotrophoblast, serving to protect the fetus from the maternal immune response [7]. In addition, HLA-G expression is detected at reduced levels in various normal adult human tissues [8]. Overexpression of HLA-G has been linked with many types of cancers, including breast, ovarian, lung, renal, gastric, and colorectal cancer, and hematological malignancies [9]. HLA-G in tumors promotes the immune-suppressive microenvironment, which results in cancer progression and poor prognosis [10]. HLA-G facilitates the escape of tumor cells from immunosurveillance by various mechanisms. It inhibits the functions of natural killer (NK) cells, CD8+ T cells, CD4+ T cells, B cells, and DC cells through interactions with specific receptors, namely immunoglobulin-like transcript 2 (ILT2/CD85j/LILRB1), immunoglobulin-like transcript 4 (ILT4/CD85d/LILRB2), and killer cell immunoglobulin-like receptor 2DL4 KIR2DL4 (CD158d) [11]. Through trogocytosis, HLA-G can transfer to T cells, leading to the production of immunosuppressive CD4+HLA-G+ and CD8+HLA-G+ T cells as well as HLA-G+ APCs, such as DC-10 and HLA-G+ macrophages [12]. Furthermore, soluble HLA-G (sHLA-G) binds to CD8 and triggers apoptosis in CD8-bearing cells through interaction with the Fas/Fas ligand molecules [13]. HLA-G can also modulate the inflammatory microenvironment of the tumors through the release of cytokines. It was shown that pro-inflammatory and Th1 cytokines such as IL-2, IL-6, and TNF downregulate HLA-G expression on JEG-3 cells; however, Th2 cytokines such as IL-4 and IL-10 upregulate its expression. IFN-γ also plays a role in the maintenance of HLA-G expression [14]. 

Genetic variants of HLA-G located in both the 5′URR (upstream regulatory region) and 3′UTR (untranslated region) are associated with different HLA-G expression levels. The HLA-G 14 bp insertion/deletion (ins/del) polymorphism (rs16375), located in the 3′UTR, belongs to the most studied polymorphisms among different diseases. The 14-base-pair (bp) insertion (ins) allele has been linked to reduced expression of the HLA-G molecule, while the 14 bp deletion (del) allele has been associated with an increase in protein expression levels [15]. Numerous studies have explored the relationship between the HLA-G 14 bp ins/del variant and various cancer risks, but the findings have been inconsistent [16]. Meta-analysis by Jiang et al. observed that the HLA-G 14 bp ins/ins genotype played a protective role in breast cancer patients when compared to the HLA-G 14 bp del/del genotype (with an odds ratio (OR) of 0.65, *p* < 0.05). Additionally, in the context of esophageal cancer, the 14 bp ins/ins genotype also exhibited a protective effect in a dominant genetic model (with an OR of 0.66, *p* < 0.05) [17].

HLA-G expression has been studied in relation to BC progression, but few studies have explored this topic thus far. The HLA-G expression has been frequently detected in bladder cell carcinoma tissues as compared to normal bladder tissues and has correlated with tumor grade [18,19]. Overexpression of HLA-G positively correlated with metastatic prostate infiltration [20]. No relationship was found by comparing the frequency of the HLA-G 14 bp polymorphism between urinary bladder cancer patients and control subjects [21]. 

Heart rate variability (HRV) is a measure of the variation in time between heartbeats. It reflects the balance between the sympathetic nervous system (fight-or-flight) and the parasympathetic nervous system (rest-and-digest). High HRV generally indicates a healthy autonomic nervous system, better stress resilience, and improved overall health. Low HRV can be associated with stress, anxiety, and various health conditions [22]. HRV and HLA-G are two distinct biological entities that, while unrelated in their primary functions, can indirectly influence each other through their roles in the immune system and stress response. Chronic stress can lead to low HRV and impaired immune responses [23]; however, its relation to HLA-G expression has not yet been studied. 

Until now, no study has evaluated the association between the HLA-G 14 bp ins/del polymorphism, HLA-G expression, and the risk of BC in the urine cells. The primary objective of this study was to analyze the correlation between the HLA-G 14 bp ins/del polymorphism, HLA-G expression, and clinical parameters in BC patients. The secondary objective was to investigate the impact of HRV parameters defining autonomic nervous regulation on HLA-G expression in BC patients.

## 2. Materials and Methods

### 2.1. Study Subjects

This study enrolled 89 BC patients (63 males and 26 females) who underwent partial or complete resection of bladder tumors. The average age of BC patients was 68.30 ± 8.83 years. Patients were randomly recruited from the Department of Urology, St. Cyril and Methodius Hospital Bratislava, Slovakia. The diagnosis was approved by two pathologists according to the most recent WHO classification criteria [24]. Blood samples were obtained from the patients on the day of surgical treatment. The reference group comprised 74 age-matched unrelated individuals (41 males and 33 females) with a mean age of 66.41 ± 10.06 years. All control subjects were without any personal or family history of cancer or acute inflammatory disease, and they were randomly recruited from a larger population sample. All participants provided written informed consent to be enrolled in this study and for personal data management. The study was conducted in accordance with the International Ethical Guidelines and the Declaration of Helsinki and was approved by the Ethical Committees of both the Faculty of Medicine, Comenius University, and University Hospital in Bratislava, Slovakia (approval code: 125/2021), and St. Cyril and Methodius Hospital, Bratislava (approval code: EK/1/2/2022).

### 2.2. Analysis of HLA-G 14 bp Ins/Del Polymorphism at the 3′UTR Region

Genomic DNA was isolated from ethylenediamine tetra-acetic acid (EDTA)-treated whole blood samples (2 mL) by a modified salting-out procedure [25]. The HLA-G 14 bp ins/del polymorphism (rs66554220) at the 3′UTR region was analyzed via PCR using the forward primer 5′GTGATGGGCTGTTTAAAGTGTCACC-3′ and the reverse primer 5′GGAAGGAATG CAGTTCAGCATGA-3′, as described by Hviid et al. [26]. A 25 μL PCR reaction mixture contained 50 ng of template DNA, 0.2 mM of each dNTP (Thermo Fisher Scientific, Waltham, MA, USA), 1 U of Taq DNA polymerase (Thermo Fisher Scientific, Waltham, MA, USA), 1.5 mmol MgCl_2_ (Thermo Fisher Scientific, Waltham, MA, USA), and 10 pmol of each specific primer. The PCR conditions consisted of initial denaturation at 94 °C for 3 min, followed by 30 cycles of denaturation at 94 °C for 1 min, annealing at 64 °C for 1 min, and elongation at 72 °C for 1 min. The final elongation at 72 °C for 10 min completed the reaction. The PCR products were run in 3.0% agarose gel for 30 min and then visualized under UV light. PCR fragments of 224 bp (14 bp insertion) and 210 bp (14 bp deletion) were detected using a 100 bp DNA ladder (Solis BioDyne, Tartu, Estonia). 

### 2.3. Real-Time RT-PCR Analysis of HLA-G Expression

Cells were isolated from 10 mL of urine samples of both BC patients and control subjects by centrifugation at 2500 rpm and resuspended in 1 mL of RNAlater solution (Qiagen, Hilden, Germany). The cell samples were stored at −70 °C until usage. Total RNA was extracted using the manufacturer’s TRIzol RNA extraction protocol (Life Technologies, Carlsbad, CA, USA). The RNA pellet was dissolved in RNase-free water and stored at −70 °C. To synthesize cDNA from total RNA, the High-Capacity RNA-to-cDNA Kit (Applied Biosystems, Waltham, MA, USA) was used. The 20 μL reaction mixture, which contained RNA at a concentration of 1 μg, underwent an initial incubation at 25 °C for 10 min, followed by a subsequent incubation at 42 °C for 60 min. The reaction was completed by heating it at 95 °C for 5 min. cDNA served as the template for HLA-G amplification. Specific primers and probes targeting HLA-G were selected to amplify all isoforms of HLA-G transcripts (HLA-G/F: 5′- CTGGTTGTCCTTGCAGCTGTAG-3′, HLA-G/R: 5′-CCTTTTCAATCTGAGCTCTTCTTTCT-3′, HLA-Gprobe: 5′-CACTGGAGCTGCGGTCGCTGCT-3′). The TaqMan probes were labeled with FAM reporter dye 6-carboxyfluorescein at the 5’ end and TAMRA quencher dye 6-carboxytetramethylrhodamine at the 3’ end. These probes recognize a sequence located between the PCR primers. For real-time PCR, a 25 μL reaction mixture contained 1x TaqMan^®^ Gene Expression Master Mix (Thermo Fisher Scientific), 200 nM of specific primers, 150 nM of a specific TaqMan probe, and 3 μL of cDNA. Following the manufacturer’s recommendations, the cDNA was amplified using the ABI Prism7000 Sequence Detection System (Applied Biosystems). The PCR began with denaturation at 95 °C for 10 min, followed by 40 cycles of denaturation at 95 °C for 15 s and annealing at 60 °C for 1 min. The glyceraldehyde-3-phosphate dehydrogenase (GAPDH) gene was expressed using specific probes and primers and served as the endogenous control (GAPDH/F: 5′-CATGGGTGTGAACCATGAGAA-3′, GAPDH/R: 5′-GGTCATGAGTCCTTCCACGAT-3′, GAPDH probe: 5′-AACAGCCTCAAGATCATCAGCAATGCCT-3′). 

Values of cycle threshold (CT) were determined via automated threshold analysis using ABI Prism software (Thermo Fisher Scientific, Version 3.7). To calculate the changes in gene expression of individual urine cell samples, the comparative cycle threshold method (2^−ΔΔCT^) was used [27]. The HLA-G expression in urine cell samples was compared to the expression in HLA-G-positive JEG-3 cells (American Type Culture Collection (ATCC), HTB-36) (expression = 1) to obtain the relative expression value. 

### 2.4. Serum sHLA-G and Cytokine Level Analysis

Serum was obtained by centrifugation of 3 mL of clotting blood samples of BC patients on the day of chirurgical treatment. Serum was obtained from healthy control subjects as well. The serum level of soluble HLA-G (sHLA-G) was determined by sandwich ELISA according to the manufacturer’s recommendation (human sHLA-G ELISA kit; BioVendor, Brno, Czech Republic). Concentrations of the anti-inflammatory cytokine IL-10, the pro-inflammatory cytokines IL-6, TNF, and IFN-γ, and the chemokine MCP-1 were also determined by sandwich ELISA assay kits (Fine Biotech Co., Ltd., Wuhan, China). 

### 2.5. Measurement of HRV Parameters

Heart rate variability determination was based on 5 min ECG measurements conducted on patients and healthy control subjects in a supine position after a 5 min supine rest. The Bittium Faros 180 electrocardiogram device (Bittium Biosignals Ltd., Oulu, Finland) was used with electrodes placed on the right first and left fifth intercostal spaces at the midclavicular line. Skin was prepared with ethanol prior to electrode placement. A sampling rate of 1000 Hz was employed to capture the electrical signal. HRV analysis was performed using the Kubios Premium system (Kubios Oy, Kuopio, Finland) to assess autonomic nervous system (ANS) balance and activity. Selected HRV parameters known to reflect sympathetic and parasympathetic activity were analyzed [28]. Indices associated with the sympathetic nervous system activity included time-domain measures of mean heart rate (HR, beats/min), modified acceleration capacity of the heart rate (ACmod, ms), the frequency-domain parameter of the normalized low-frequency band (LF, n.u.), the sympathetic nervous system (SNS) index, and the stress index. Vagally mediated HRV parameters included the time-domain parameters of the standard deviation of normal-to-normal interbeat intervals (SDNN, ms), root mean square of successive differences between normal heartbeats (RMSSD, ms), modified deceleration capacity of the heart rate (DCmod, ms), frequency-domain parameters of the normalized high-frequency band (HF, n.u.), and the parasympathetic nervous system (PNS) index. Other analyzed parameters were the total power and the sum of the energy in the LF, HF, and very-low-frequency (VLF) bands for short-term recordings, reflecting overall autonomic activity and current person adaptability.

### 2.6. Statistical Analysis

For statistical analysis, the programs InStat GraphPad (San Diego, CA, USA, version 3.10) and SAS Enterprise Guide (Cary, NC, USA, version 6.1) were used. We determined the allele and genotype frequencies of the HLA-G 14 bp ins/del polymorphism by direct counting. We then assessed whether the genotypes deviated from Hardy–Weinberg equilibrium (HWE) using the chi-squared goodness-of-fit test. Additionally, we employed the Pearson chi-squared statistical test to compare the HLA-G 14 bp ins/del allele and genotype frequencies between the two groups under study (BC patients vs. control cohort) (InStat GraphPad Software, version 3.10). The *p*-values and odds ratios (ORs) with 95% confidence intervals (CIs) were subjected to analysis in co-dominant, dominant, recessive, and over-dominant inheritance models. The multivariate logistic regression analysis adjusting for age and sex as possible influencing factors was performed using SNPstats web software available at https://www.snpstats.net/start.htm (accessed on 15 February 2024) [29]. Mann–Whitney test was used to investigate the differences between relative HLA-G expression or sHLA-G serum levels and the selected variables including tumor grade, disease stage, and body mass index (BMI). The Spearman′s correlation method was used to analyze the correlation of relative HLA-G mRNA expression or sHLA-G and cytokine serum levels with HRV parameters. The patient population was dichotomized by the healthy range of sHLA-G into low- and high-sHLA-G groups. The groups were compared using the log-rank test. Kaplan–Meier analysis was used for the estimation of disease-free survival (DFS). DFS was the time period from patient enrollment to disease recurrence, last follow-up, or death from any potential cause. The results were expressed as the median and interquartile range (IQR), or mean ± standard deviation (SD). A *p*-value of less than 0.05 was considered statistically significant. 

## 3. Results

### 3.1. Characteristics of the Study Groups

The baseline characteristics of the study population are shown in Table 1. This study included 89 BC patients and 74 control subjects. The difference between the BC group and controls in relation to gender was not statistically significant (*p* = 0.06). Males had a higher prevalence in both BC patients (70.79%) and control subjects (55.41%). No statistically significant difference between the studied groups was found in relation to age at examination (*p* = 0.23). Out of 89 BC patients, 42 patients were diagnosed with low-grade tumors and 47 with high-grade tumors. According to the type of UC invasion, the patients were grouped into NMIBC with pTa/CIS (*n* = 37) and pT1 (*n* = 31) tumors and MIBC with pT2 (*n* = 17) and pT3 (*n* = 4) tumors. The patients underwent either transurethral resection of bladder tumor (TURBT, *n* = 77) or radical cystectomy (*n* = 12). The majority of BC patients had a positive history of smoking (56 vs. 33). Mean DFS time was 9.87 ± 7.89 months. 

### 3.2. Analysis of HLA-G 14 bp Ins/Del Polymorphism with HLA-G mRNA Expression and Serum sHLA-G Levels in BC Patients and Control Group

The association between the HLA-G 14 bp ins/del polymorphism and BC risk is shown in Table 2. The genotype distribution fits the HWE in BC patients (χ^2^ = 0.08, *p* = 0.78) as well as in control subjects (χ^2^ = 0.48, *p* = 0.49). No statistically significant differences in the HLA-G 14 bp ins/del allele (*p* = 0.39, OR = 0.52−1.25) and genotype (*p* > 0.05, OR = 0.63–1.20) distribution under four genetic models were determined between BC patients and the control subjects. Multivariate analysis of associations between the 14 bp ins/del polymorphism and BC risk adjusted for age and sex revealed no changes in comparison with the univariate analysis (*p* > 0.05, OR = 0.63−1.15, Table 2). 

Correlation analysis of the HLA-G 14 bp ins/del polymorphism with relative HLA-G mRNA expression or serum sHLA-G levels was performed in BC patients and control subjects. The carriers of HLA-G 14 bp ins/ins genotypes under the recessive genetic model showed a significantly lower relative HLA-G mRNA expression as compared to other genotype carriers in the BC group (0.019 ± 0.025 vs. 1.5041 ± 1.9302, *p* = 0.049). However, in the control group, no statistically significant difference between HLA-G 14 bp ins/del genotypes and relative HLA-G mRNA expression was found (*p* > 0.05, Table 3). Following sHLA-G analysis, the carriers of HLA-G 14 bp del/del and ins/del genotypes showed a significantly higher sHLA-G serum level as compared to 14 bp ins/ins genotype carriers in the BC group (*p* = 0.03, Table 4). 

### 3.3. Association of HLA-G mRNA Expression and sHLA-G Serum Levels with Tumor and Clinical Variables in BC Patients

An analysis of the association between HLA-G mRNA expression and sHLA-G serum levels and the selected variables including grade, stage, and BMI was performed in BC patients. The results of HLA-G mRNA expression analysis revealed no significant differences between BC patients and control subjects (1.312 ± 1.867 vs. 1.333 ± 1.539, *p* = 0.2938). There was also no statistically significant association of relative HLA-G mRNA expression with low-grade and high-grade (0.938 ± 1.359 vs. 1.746 ± 2.276, *p* = 0.3270, Table 5) tumors. The analysis of disease stage found significantly higher relative HLA-G mRNA expression in patients with pT2 and pT3 as compared to patients with pTa/CIS and pT1 (2.101 ± 2.008 vs. 1.086 ± 1.786, *p* = 0.0436, Table 5). In the subgroup of smokers, there was only a tendency towards an increase in relative HLA-G mRNA expression in the pT2 and pT3 stages as compared to the pTa and pT1 stages (2.312 ± 2.139 vs. 1.116 ± 2.022, *p* = 0.0583, Table 5). Concerning HLA-G mRNA expression related to grade and stage, there was significantly higher relative HLA-G mRNA expression in high-grade MIBCs as compared to low-grade NMIBCs (2.101 ± 2.008 vs. 0.938 ± 1.359, *p* = 0.0365, Table 5). Subgroup analysis related to BMI revealed no statistically significant association of HLA-G mRNA expression with BMI (*p* > 0.05, Table 5). The Kaplan–Meier analysis showed no association between HLA-G mRNA expression and DFS (*p* > 0.05). 

**Figure 1:**
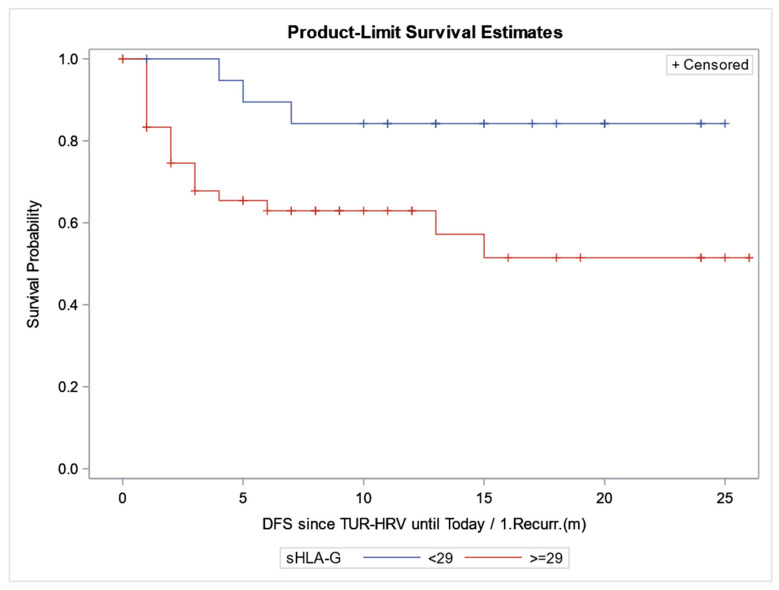
Kaplan–Meier estimates of disease-free survival according to 29 U/mL sHLA-G levels. X-axis: disease-free survival (months); Y-axis: survival probability (*p* = 0.038). Number 1 (blue line): sHLA-G levels less than 29 U/mL; Number 2 (red line): sHLA-G levels more than 29 U/mL. DFS: disease-free survival; HRV: heart rate variability; m: months; Recurr: recurrence; TUR: transurethral resection. + Censored means TUR-censored data.

sHLA-G serum level analysis revealed no significant differences between BC patients and control subjects (36.812 ± 16.591 vs. 35.627 ± 8.883, *p* = 0.4798). There was also no significant association of sHLA-G serum levels with tumor grade and disease stage (*p* > 0.05, Table 6). However, a Kaplan–Meier survival analysis revealed that patients with sHLA-G serum levels less than 29 U/mL have significantly higher DFS as compared to patients with serum levels above 29 U/mL (*p* = 0.038, HR: 0.35, 95% CI: 0.15–0.81, Figure 1). 

### 3.4. Correlation of sHLA-G with IL-6, IL-10, TNF, IFN-γ, and MCP-1 Levels in BC Patients

Spearman’s rank correlation between sHLA-G and IL-6, IL-10, TNF, IFN-γ, and MCP-1 levels was calculated. The results showed that the sHLA-G positively significantly correlated with the levels of the pro-inflammatory cytokine IL-6 (*p* = 0.0088, Table 7). However, we did not find a correlation of sHLA-G with the levels of the pro-inflammatory cytokines TNF and IFN-gamma, anti-inflammatory cytokine IL-10, and the chemokine MCP-1 (*p* > 0.05, Table 7). 

### 3.5. Correlation of sHLA-G Serum Levels with Heart Rate Variability Parameters 

**Figure 2:**
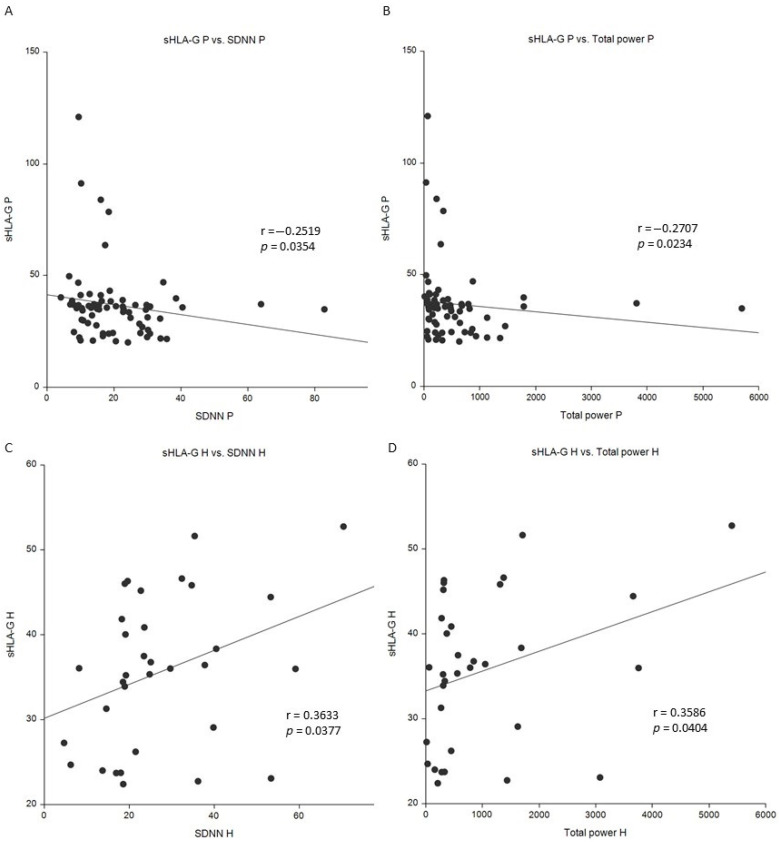
Correlation of sHLA-G serum level with SDNN and total power in BC patients and control group. sHLA-G serum level vs. SDNN in BC patients (**A**) and control subjects (**C**), sHLA-G serum level vs. total power in BC patients (**B**) and control subjects (**D**). H: healthy; P: patients; SDNN: standard deviation of normal-to-normal interbeat intervals (ms); sHLA-G: soluble HLA-G level (U/mL); total power: the sum of the energy in the low-frequency, high-frequency, and very-low-frequency range (ms^2^). Spearman r and *p*-values are displayed. A *p*-value < 0.05 was considered statistically significant. The line in the figure illustrates the fitted curve for Spearman′s correlation method.

In the study patient population, sHLA-G serum levels significantly negatively correlated with parasympathetic HRV parameters such as SDNN (standard deviation of NN intervals) (*p* = 0.0354, Figure 2A), pNNxx (number of successive RR interval pairs that differ more than xx ms divided by the total number of RR intervals) (*p* = 0.0407), TINN (baseline width of the RR interval histogram) (*p* =0.0355), and total power (ms^2^) (*p* = 0.0234, Figure 2B). Within the carriers of the at-risk HLA-G 14 bp del/del and 14 bp ins/del genotype subgroup, there was a negative correlation of sHLA-G serum level with pNNxx (*p* = 0.0285) and PNS index (*p* = 0.0241) and a positive correlation with mean HR (beats/min) (*p* = 0.0446) and SNS index (*p* = 0.0407, Table 8). 

However, in the control group, we observed opposite correlations in comparison to BC patients. sHLA-G serum levels significantly positively correlated with parasympathetic HRV parameters such as SDNN (ms) (*p* = 0.0377, Figure 2C), RMSSD (ms) (*p* = 0.0182), DCmod (ms) (*p* = 0.0109), and total power (ms^2^) (*p* = 0.0404, Figure 2D), and negatively correlated with sympathetic HRV parameters such as ACmod (ms) (*p* = 0.0097) and stress index (*p* = 0.0486) (Table 8). 

## 4. Discussion

HLA-G is a non-classical HLA antigen (class Ib) with immunosuppressive effects. In tumors, HLA-G mediates the suppressive microenvironment, affecting both innate and adaptive immune responses [30]. A positive correlation between HLA-G expression and advanced disease stage, tumor metastasis, poor prognosis, and shorter DFS has been reported in many cancer types [10]. It is well established that the expression level of HLA-G is influenced by various genetic polymorphisms. Among these, the most significant polymorphism is the 14-base-pair insertion/deletion (14 bp ins/del) variant (rs66554220), which is located in the 3’ untranslated region (3’ UTR). As the HLA-G 14 bp insert in the 3′UTR region is associated with decreased HLA-G expression [15], we evaluated the HLA-G 14 bp ins/del allele and genotype frequencies of 89 BC patients and compared them with 78 control subjects. We found no association between the HLA-G 14 bp ins/del polymorphism and BC risk. This is in agreement with the study by Castelli et al. [21], which also revealed no correlation between the HLA-G 14 bp polymorphism and BC risk. Recent meta-analyses reported that the HLA-G 14 bp Ins/Ins genotype and Ins allele are linked to a reduced breast and esophageal cancer risk [17,31]. 

We also evaluated the correlation between the HLA-G 14 bp ins/del polymorphism and relative HLA-G mRNA expression and/or serum sHLA-G levels in both BC patients and control subjects. We analyzed the HLA-G mRNA expression in the cells of the urine sediment, which has not been reported until now. Our results revealed that carriers of HLA-G 14 bp ins/ins genotypes under the recessive genetic model had a significantly lower relative HLA-G mRNA expression level as compared to other genotype carriers in the BC group. As the HLA-G 14 bp insertion variant in the 3′ UTR is linked with decreased HLA-G expression, this allele should be considered protective among other alleles in BC progression. A previous study on HLA-G alleles in urinary bladder TCC patients found that the HLA-G∗0104 group was linked to the progression of high-grade tumors, regardless of smoking habits. In contrast, the G∗0103 allele was associated with high-grade tumors only in smoking patients [21]. The 14 bp deletion allele has been associated with the stage of breast cancer [32]; however, this allele has been associated with a decreased risk of invasive cervico-vaginal cancer in smokers [33]. 

Analyses of the associations between local HLA-G mRNA expression in tumor urine cells and/or soluble HLA-G serum levels and the selected variables including tumor grade, disease stage, and BMI were performed in BC patients. Our results revealed significantly higher relative HLA-G mRNA expression in pT2 and pT3 as compared to patients with pTa/CIS and pT1. In the smoking BC patients, a significant association of relative HLA-G mRNA expression with disease stage was also found. Finally, there was a significant high increase in the mRNA expression of HLA-G in the high-grade MIBC group as compared to the low-grade NMIBC group. Our results partially agree with Moavro et al. [19], who reported increased HLA-G staining levels in the bladder UC tissues of high-grade as compared to low-grade BC. In contrast, other studies reported no significant association of HLA-G expression with tumor grade, stage, schistosomiasis, and lymph node involvement [18,20]. However, there was a highly significant increase in the expression of HLA-G in bladder cancer cases with metastatic prostate infiltration [20]. The positive correlation of HLA-G expression in tumor lesions with disease stage has been reported for other cancer types including breast cancer [34], cervical cancer [35,36], colorectal cancer [37], pancreatic carcinoma [38], renal cell carcinoma [39], and esophageal squamous cell carcinoma [40]. 

The sHLA-G levels in the serum of our BC patients and healthy control individuals did not differ as reported by others [18]. In our study, no significant associations of sHLA-G serum levels with tumor grade and disease stage were found in BC patients.

We also evaluated the associations of HLA-G mRNA expression and sHLA-G serum levels with DFS in BC patients. As the previously reported median values of sHLA-G levels in healthy control subjects were in the range of 23 to 35 U/mL, we chose a cut-off value of 29 U/mL for predicting DFS probability in BC patients [41,42]. The Kaplan–Meier analysis revealed that patients with sHLA-G serum levels less than 29 U/mL have a significantly higher DFS as compared to patients with serum levels above 29 U/mL. Jiao et al. [43] reported that the cut-off value of sHLA-G for predicting the five-year survival of colorectal cancer patients was 50.8 U/mL. The Kaplan–Meier survival curve showed that the survival prognosis of patients in the low-level group was significantly better than that in the high-level group [43]. The other results using the Kaplan–Meier Plotter database showed that patients with higher HLA-G mRNA expression in pancreatic ductal adenocarcinoma and gliomas had significantly shorter DFS [44]. In contrast, higher HLA-G levels in the bladder UC, renal clear cell carcinoma, renal cell carcinoma, hepatocellular carcinoma, lung adenocarcinoma, and ovarian carcinoma groups were significantly associated with longer DFS [44,45]. Furthermore, higher sHLA-G levels in chronic myeloid leukemia with the G*01:01:01 or G*01:01:02 allele showed shorter DFS [46]. HLA-G expression determined by immunostaining was shown to significantly correlate with reduced DFS in the subgroup of CRC-related deaths [47,48]. 

As inflammation represents one of the pathological events associated with cancer development, we also analyzed the impact of sHLA-G on cytokine levels in BC patients. sHLA-G significantly positively correlated with the pro-inflammatory cytokine IL-6. However, we did not find a correlation of sHLA-G with the level of the pro-inflammatory cytokines TNF and IFN-γ, anti-inflammatory cytokine IL-10, and chemokine MCP-1. It has been reported that pro-inflammatory and Th1 cytokines such as IL-2, IL-6, and TNF downregulate HLA-G expression in JEG-3 cells; however, Th2 cytokines such as IL-4 and IL-10 upregulate its expression. IFN-γ also plays a role in the maintenance of HLA-G expression [14]. Elevated IL-6 expression has been shown to stimulate tumor growth and is associated with metastasis in the majority of cancer cases [49]. The role of IL-6 in HLA-G expression has not been clearly identified. It was reported that IL-6 exerts anti-inflammatory effects as well. IL-6 enhances the release of IL-10 and IL-1RA from macrophages, suppresses TNFα release from monocytes, decreases macrophage infiltration in adipose tissue, and helps the polarization of macrophages toward the anti-inflammatory M2 phenotype [50]. As IL-10 induces the expression of HLA-G, a positive correlation between HLA-G and IL-6 levels in some pathological conditions can be hypothesized. 

It is known that the autonomic nervous system can also modulate the activity of the immune system, and particularly the vagal component is associated with the cholinergic anti-inflammatory pathway [51]. Several studies have reported a correlation between reduced HRV and cancer progression and outcome [52,53]. The latest meta-analysis by Adam et al. found a significant association of a pro-inflammatory state with reduced overall variability indexed by total power HRV [54]. In this study, we investigated the impact of HRV parameters on sHLA-G in BC patients and control subjects. In BC patients, there was a negative correlation between sHLA-G serum levels and HRV parameters associated with parasympathetic modulation, while in healthy individuals, this correlation was positive. Moreover, in the group of HLA-G 14 bp del risk allele carriers, besides a negative association of HLAG levels with parasympathetic HRV indices, a positive correlation with sympathetic HRV indices was also observed. 

The reduced parasympathetic activity seen in BC patients associated with elevated sHLA-G levels implies that chronic stress and autonomic dysregulation in the BC group might trigger higher sHLA-G production, potentially also contributing to immune system suppression and disease progression in BC patients [9]. A balanced HRV profile with dominant parasympathetic activity might underlie the normal regulatory HLA-G function in healthy individuals [55]. This suggests that a balanced autonomic nervous system could play a protective role by maintaining sHLA-G function in the healthy range.

As HLA-G expression is upregulated in BC patients, HLA-G and its receptors represent potential targets for cancer immunotherapy. The promising strategy includes targeting the HLA-G/ILT signaling pathway alongside the use of CTLA-4/B7 and PD-1/PD-L1 as new immune checkpoints in cancer development [56]. The association of enhanced expression of ILT-2 and ILT-4 receptors with malignancy and poor prognosis has been reported in many previous studies [57,58]. Regarding BC, the levels of peripheral CD8+ILT2+ T cells have been analyzed to predict NMIBC recurrence. There was a strong association between CD8+ILT2+ T-cell population levels and recurrence. Among patients, the two-year recurrence-free survival rates were 83% for those with less than 18% CD8+ILT2+ T cells, 39% for the intermediary group, and 12% for patients with more than 46% CD8+ILT2+ T cells [59]. It was found that the tumor-infiltrating CD8+ ILT2+ T cells’ cytotoxic functions could be selectively inhibited by HLA-G expression by tumor cells. The administration of HLA-G antibodies has both enhanced CD8+ILT2+ T-cell activity and counteracted the immunosuppressive impact of HLA-G [60]. These findings, together with our results, indicate that a combination of immunotherapy approaches, such as blocking HLA-G and ILTs, could improve the clinical outcomes for patients with BC. 

## 5. Conclusions

This study analyzed the relative HLA-G mRNA expression in urine cell samples and sHLA-G serum levels and correlated them with the HLA-G 14 bp ins/del polymorphism and the selected variables including tumor grade, disease stage, BMI, and HRV parameters in BC patients. Our results revealed that the carriers of protective HLA-G 14 bp ins/ins genotypes under the recessive genetic model had a significantly lower relative HLA-G mRNA expression level as compared to other genotype carriers. Significantly higher HLA-G mRNA expression was noted in pT2+pT3 tumors as compared to those with pTa+pT1. Furthermore, higher HLA-G mRNA expression was observed in high-grade MIBCs than in low-grade NMIBCs. We also revealed a negative correlation of sHLA-G levels with DFS probability. The HRV profile with reduced parasympathetic activity was associated with elevated sHLA-G levels. Our data suggest a possible role of increased HLA-G expression in contributing to UC progression and prognosis.

## Figures and Tables

**Table 1:** Characteristics of the studied groups.

Parameter	Bladder Cancer	Control Subjects	*p*-Value
	(n = 89)	(n = 74)	
Gender ratio males/females	63/26	41/33	0.06
Age at examination (mean ± SD, years)	68.30 ± 8.83	66.41 ± 10.06	0.23
Smoking			
No	33	52	
Yes	56	22	<0.0001
BMI	27.88 ± 4.50	27.01 ± 3.32	0.21
Grade			
Low	42	-	-
High	47	-	-
Tumor staging			
pTa/CIS	37	-	-
pT1	31	-	-
pT2	17	-	-
pT3	4	-	-
DFS (mean ± SD, months)	9.87 ± 7.89	-	-
Low grade	10.14 ± 9.00	-	-
High grade	9.67 ± 7.08	-	-

Data are shown as the mean with standard deviation. n, number; SD, standard deviation. Age differences between the two groups were examined using the Mann–Whitney test. Differences in clinical variables were assessed using the χ^2^ test. A *p*-value < 0.05 was considered statistically significant.

**Table 2:** HLA-G 14 bp ins/del allele and genotype frequencies in BC patients and control subjects.

SNP/ Model	Allele/ Genotype	BC Patients (*n* = 89)	Control Subjects (*n* = 74)	Univariate Analysis		Multivariate Analysis	
				*p*-Value	OR (95%CI)	*p*-Value	OR (95%CI)
14 bp ins/del	14 del	101 (56.74%)	76 (51.35%)				
	14 ins	77 (43.26%)	72 (48.65%)	0.39	0.80 (0.52−1.25)	-	-
	14 del/del	28 (31.46%)	21 (28.38%)		1.00		1.00
Co-dominant	14 ins/del	45 (50.56%)	34 (45.94%)	0.49	0.99 (0.48−2.04)	0.54	0.95 (0.45−1.97)
	14 ins/ins	16 (17.98%)	19 (25.68%)		0.63 (0.26−1.51)		0.63 (0.26−1.54)
	14 del/del	28 (31.46%)	21 (28.38%)		1.00		1.00
Dominant	14 ins/del + 14 ins/ins	61 (68.54%)	53 (71.62%)	0.67	0.86 (0.44−1.70)	0.61	0.84 (0.42−1.66)
	14 del/del + 14 ins/del	73 (82.02%)	55 (74.32%)		1.00		1.00
Recessive	14 ins/ins	16 (17.98%)	19 (25.68%)	0.23	0.63 (0.30−1.35)	0.27	0.65 (0.30−1.40)
	14 del/del + 14 ins/ins	44 (49.44%)	40 (54.06%)		1.00		1.00
Over-dominant	14 ins/del	45 (50.56%)	34 (45.94%)	0.56	1.20 (0.65−2.23)	0.67	1.15 (0.61−2.16)
HWE (χ^2^/*p*)		0.08/0.78	0.48/0.49				

Allele and genotype frequencies are presented as absolute numbers with percentages in parentheses. BC, bladder cancer; CI, confidence interval; HWE, Hardy–Weinberg equilibrium; n, number; OR, odds ratio. Univariate analysis is based on the χ^2^ test. Multivariate analysis is adjusted by sex and age. A *p*-value < 0.05 was considered statistically significant.

**Table 3:** Association of HLA-G 14 bp ins/del genotypes with HLA-G mRNA expression in BC patients and control subjects.

Parameter	14 bp del/del	14 bp ins/del	14 bp ins/ins	p/p* CM	p/p* DM	p/p* RM	p/p* OD
relative HLA-G mRNA expression in BC group	1.587 ± 2.059 (n = 20)	1.443 ± 1.867 (n = 27)	0.019 ± 0.025 (n = 7)	0.14/0.14	0.41/0.42	**0.049/0.049**	0.61/0.62
relative HLA-G mRNA expression in control subjects	1.520 ± 1.293 (n = 8)	1.519 ± 1.802 (n = 15)	0.799 ± 1.240 (n = 8)	0.54/0.38	0.70/0.72	0.26/0.16	0.53/0.39

BC, bladder cancer; CM, co-dominant model; del, deletion; DM, dominant model; ins, insertion; n, number; OD, over-dominant model; RM, recessive model; SD, standard deviation. The *p*-value < 0.05 was considered statistically significant and is marked in bold. *p—*p*-value adjusted for sex and age.

**Table 4:** Association of HLA-G 14 bp ins/del genotypes with sHLA-G serum levels in BC patients and control subjects.

Parameter	14 bp del/del	14 bp ins/del	14 bp ins/ins	p/p* CM	p/p* DM	p/p* RM	p/p* OD
sHLA-G level in BC group	31.008 ± 7.723 (n = 25)	40.762 ± 21.293 (n = 32)	38.249 ± 13.349 (n = 13)	0.08/0.09	**0.03/0.03**	0.73/0.76	0.07/0.06
sHLA-G level in control subjects	39.024 ± 10.049 (n = 11)	35.938 ± 8.995 (n = 12)	31.515 ± 6.035 (n = 10)	0.15/**0.03**	0.12/0.07	0.08/**0.01**	0.88/0.80

BC, bladder cancer; CM, co-dominant model; del, deletion; DM, dominant model; ins, insertion; n, number; OD, over-dominant model; RM, recessive model; SD, standard deviation; sHLA-G, soluble HLA-G. The *p*-value < 0.05 was considered statistically significant and is marked in bold. *p—*p*-value adjusted for sex and age.

**Table 5:** Association of HLA-G mRNA expression with clinical characteristics in BC patients.

Clinical Parameter	Relative HLA-G mRNA Expression (Mean ± SD)	*p*-Value	Median, IQR
**Grade**			
Low grade (n = 29)	0.938 ± 1.359		0.3194; 1.3325
High grade (n = 25)	1.746 ± 2.276	0.3270	0.4156; 3.3750
**Tumor stage**			
pTa+pT1 (n = 42)	1.086 ± 1.786		0.2790; 1.3497
pT2+pT3 (n = 12)	2.101 ± 2.008	**0.0436**	1.199; 3.6500
**Tumor stage in smokers**			
pTa+pT1 (n = 23)	1.116 ± 2.022		0.1952; 1.3384
pT2+pT3 (n = 9)	2.312 ± 2.139	0.0583	1.857; 3.9665
**Grade-Stage**			
I. Low-grade NMIBC (n = 29)	0.938 ± 1.359	0.6633 (I vs. II)	0.3194; 1.3325
II. High-grade NMIBC (n = 13)	1.418 ± 2.534	0.2051 (II vs. III)	0.1270; 1.2586
III. High-grade MIBC (n = 12)	2.101 ± 2.008	**0.0365** (I vs. III)	1.199; 3.6500
**Body mass index**			
I: 18 to 24.9 (n = 11)	1.463 ± 1.598	0.2956 (I vs. II)	0.4127; 1.9561
II: 25 to 29.9 (n = 27)	1.467 ± 2.123	0.5382 (II vs. III)	0.3905; 1.5104
III: 30 to 40 (n = 16)	0.9450 ± 1.613	0.1524 (I vs. III)	0.3425; 0.6611

*p*-value assessed using the Mann–Whitney test. IQR, interquartile range; MIBC, muscle-invasive bladder cancer; n, number; NMIBC, non-muscle-invasive bladder cancer; SD, standard deviation. A *p*-value < 0.05 was considered statistically significant and is marked as bold.

**Table 6:** Association of sHLA-G serum levels with clinical characteristics in BC patients.

Clinical Parameter	sHLA-G Level (Mean ± SD)	*p*-Value	Median, IQR
**Grade**			
Low grade (n =29 )	35.201 ± 8.490		35.113; 8.8041
High grade (n = 41)	37.952 ± 20.523	0.5159	35.602; 12.7473
**Tumor stage**			
pTa+pT1 (n = 54)	37.512 ± 16.846		35.620; 8.1391
pT2+pT3 (n = 16)	34.448 ± 15.994	0.2373	31.456; 13.9493
**Tumor stage in smokers**			
pTa+pT1 (n = 33)	40.915 ± 14.386		36.128; 4.7208
pT2+pT3 (n = 11)	35.553 ± 18.257	0.3160	32.184; 15.9123
**Grade-Stage**			
I. Low-grade NMIBC (n = 29)	35.201 ± 8.490	0.9240 (I vs. II)	35.113; 8.8041
II. High-grade NMIBC (n = 25)	40.194 ± 22.993	0.3428 (II vs. III)	35.752; 6.9149
III. High-grade MIBC (n = 16)	34.448 ± 15.994	0.2502 (I vs. III)	31.456; 13.9493
**Body mass index**			
I: 18 to 24.9 (n = 21)	33.516 ± 13.401	0.2753 (I vs. II)	34.737; 12.4575
II: 25 to 29.9 (n = 31)	37.498 ± 17.788	0.7875 (II vs. III)	35.602; 7.9045
III: 30 to 40 (n = 18)	39.476 ± 18.052	0.2000 (I vs. III)	35.244; 12.2573

*p*-value assessed using the Mann–Whitney test. BC, bladder cancer; IQR, interquartile range; MIBC, muscle-invasive bladder cancer; n, number; NMIBC, non-muscle-invasive bladder cancer; SD, standard deviation. A *p*-value < 0.05 was considered statistically significant.

**Table 7:** Correlation of sHLA-G with IL-6, IL-10, TNF, IFN- γ, and MCP-1 in BC patients.

sHLA-G (U/mL)	Spearman r	95% CI	*p*-Value
sIL-6 (n = 70)	0.3110	0.0749 to 0.5141	**0.0088**
sIL-10 (n = 59)	0.0548	−0.2117 to 0.3136	0.6804
sTNF (n = 69)	0.1610	−0.0858 to 0.3892	0.1862
sIFN-gama (n = 59)	0.0399	−0.2258 to 0.3001	0.7642
sMCP-1 (n = 59)	0.0415	−0.2243 to 0.3016	0.7550
IFN-γ/IL-10 (n = 59)	−0.0666	−0.3243 to 0.2002	0.6161

*p*-value calculated by Spearman rank correlation. BC, bladder cancer; 95% CI, 95% confidence interval; n, number; sHLA-G, soluble HLA-G. A *p*-value < 0.05 was considered statistically significant and is marked as bold.

**Table 8:** Association of sHLA-G serum levels with HRV characteristics in BC patients and control subjects.

HRV Parameter	BC Patients		Control Subjects	
	Spearman r	*p*-Value	Spearman r	*p*-Value
SDNN (ms)	−0.2519	**0.0354**	0.3633	**0.0377**
pNNxx (%)	−0.24531	**0.0407**	0.40828	**0.0183**
	−0.2929*	**0.0285** *		
TINN (ms)	−0.25173	**0.0355**	0.34178	**0.0516**
Mean HR (beats/min)	0.1399 0.2695 *	0.2478 **0.0446** *	−0.1324	0.4628
RMSSD (ms)	−0.1776	0.1413	0.4088	**0.0182**
DCmod (ms)	−0.1757	0.1457	0.4375	**0.0109**
ACmod (ms)	0.1683	0.1638	−0.4435	**0.0097**
Total power (ms^2^)	−0.2707	**0.0234**	0.3586	**0.0404**
PNS index	−0.1896 −0.3012 *	0.1160 **0.0241** *	0.2513	0.1583
SNS index	0.2327 0.2744 *	0.0526 **0.0407** *	−0.3269	0.0634
Stress index	0.2289	0.0567	−0.3459	**0.0486**

ACmod (ms): modified acceleration capacity of the heart rate; BC: bladder cancer; DCmod (ms): modified deceleration capacity of the heart rate, HRV: heart rate variability; mean HR: mean heart rate (beats/min); PNS index: parasympathetic nervous system; RMSSD (ms): root mean square of successive differences between normal heartbeats; SDNN: standard deviation of normal-to-normal interbeat intervals; SNS index: sympathetic nervous system index; stress index: Baevsky’s stress index, a measure of HRV reflecting cardiovascular system stress; total power: the sum of the energy in the low-frequency, high-frequency, and very-low-frequency ranges. * HLA-G 14 bp del/del and ins/del genotype carriers. A *p*-value < 0.05 was considered statistically significant and is marked in bold.

## Data Availability

The presented data are available on request from the corresponding author.
